# Naringenin Attenuates Non-Alcoholic Fatty Liver Disease by Enhancing Energy Expenditure and Regulating Autophagy via AMPK

**DOI:** 10.3389/fphar.2021.687095

**Published:** 2021-06-07

**Authors:** Ying Yang, Yue Wu, Jie Zou, Yu-Hao Wang, Meng-Xia Xu, Wei Huang, Dao-Jiang Yu, Li Zhang, Yuan-Yuan Zhang, Xiao-Dong Sun

**Affiliations:** ^1^ West China School of Basic Medical Sciences and Forensic Medicine, Sichuan University, Chengdu, China; ^2^ Department of Endocrinology, Affiliated Hospital of Southwest Medical University, Luzhou, China; ^3^ Department of Plastic Surgery, The Second Affiliated Hospital of Chengdu Medical College, China National Nuclear Corporation 416 Hospital, Chengdu, China; ^4^ Analytical and Testing Center, Sichuan University, Chengdu, China

**Keywords:** non-alcoholic fatty liver disease, naringenin, energy expenditure, autophagy, mitochondrial biogenesis

## Abstract

**Background:** The prevalence of non-alcoholic fatty liver disease (NAFLD) keeps growing recently.

**Purpose:** To investigate the effects and mechanisms of naringenin (NAR) on NAFLD.

**Methods:** High-fat diet (HFD)-induced NAFLD rats were orally administered with NAR at 10, 30, and 90 mg/kg for 2 weeks. The serum level of triglyceride (TG), total cholesterol (TC), glutamic-oxaloacetic transaminase (AST), and glutamic-pyruvic transaminase (ALT) was measured. The hepatic histology was detected by H&E and oil red O staining. L02 and Huh-7 cells were induced by sodium oleate to establish a NAFLD cell model. The effects of NAR on lipid accumulation were detected by oil red O staining. The glucose uptake and ATP content of 3T3-L1 adipocytes and C2C12 myotubes were measured. The expression of proteins of the AMPK signaling pathway in 3T3-L1 adipocytes and C2C12 myotubes was assessed by Western blotting. The mitochondrial biogenesis of 3T3-L1 adipocytes and C2C12 myotubes was measured by mitotracker orange staining and Western blotting. The biomarkers of autophagy were detected by Western blotting and immunofluorescence. The binding of NAR to AMPKγ1 was analyzed by molecular docking. Chloroquine and compound C were employed to block autophagic flux and AMPK, respectively.

**Results:** NAR alleviated HFD-induced NAFLD in rats at 10, 30, and 90 mg/kg. NAR attenuated lipid accumulation in L02 and Huh-7 cells at 0.7, 2.2, 6.7, and 20 μM. NAR increased glucose uptake, decreased the ATP content, activated the CaMKKβ/AMPK/ACC pathway, and enhanced the mitochondrial biogenesis in 3T3-L1 adipocytes and C2C12 myotubes. NAR increased autophagy and promoted the initiation of autophagic flux in 3T3-L1 preadipocytes and C2C12 myoblasts, while it inhibited autophagy in NAFLD rats, 3T3-L1 adipocytes, and C2C12 myotubes. Molecular docking showed that NAR binds to AMPKγ1. Compound C blocked effects of NAR on lipid accumulation and autophagy in L02 cells.

**Conclusion:** NAR alleviates NAFLD by increasing energy expenditure and regulating autophagy via activating AMPK directly and indirectly. The direct binding of NAR and AMPKγ1 needs further validation.

## Introduction

Non-alcoholic fatty liver disease (NAFLD) is the leading cause of chronic liver disease, which global prevalence has reached 24% and keeps rising due to the increasing prevalence of obesity worldwide (Friedman et al., 2018). NAFLD includes simple steatosis and non-alcoholic steatohepatitis, which may progress to cirrhosis and hepatocellular carcinoma. Patients with NAFLD are at higher risk of metabolic comorbidities such as cardiovascular diseases, type II diabetes mellitus (T2DM), and chronic kidney disease (Younossi et al., 2018).

NAFLD results from chronic energy imbalance, which is characterized by excessive fatty acid flux. Increasing energy expenditure is an effective strategy to combat NAFLD (Silvestri et al., 2011; Rui, 2014). The development of NAFLD involves several organs such as the liver, skeletal muscle, and adipose tissue (Watt et al., 2019). Lipid accumulation in skeletal muscle leads to insulin resistance (IR) which significantly contributes to NAFLD (Boden, 2006). Adipose tissue dysfunction and adverse alterations in glucose, fatty acid, and lipoprotein metabolism impact the regulation of *de novo* lipogenesis and lead to NAFLD (Sanders and Griffin, 2016). Considering this, our study employed hepatic cells (L02 and Huh-7 cells), 3T3-L1 preadipocytes, 3T3-L1 adipocytes, C2C12 myoblasts, and C2C12 myotubes.

AMP-activated protein kinase (AMPK) is a key energy sensor and metabolic homeostasis regulation center in mammalian cells (Smith et al., 2016). When AMPK is activated, it maintains the energy balance by regulating the intake and utilization of nutrients (Long and Zierath, 2006). Mitochondria play important roles in NAFLD by regulating energy expenditure (Bratic and Trifunovic, 2010). Enhancing mitochondrial biogenesis improves mitochondrial quality and energy expenditure (Wang S.-W. et al., 2020). Autophagy is effective in degrading lipid droplets in liver cells (Allaire et al., 2019).

The most recommended treatment for NAFLD is weight loss by improving lifestyle and dietary (Friedman et al., 2018). Although vitamin E and insulin-sensitizing agents showed some efficacy, no specific pharmacological intervention has been approved by the American Food and Drug Administration, yet.

**Figure:**
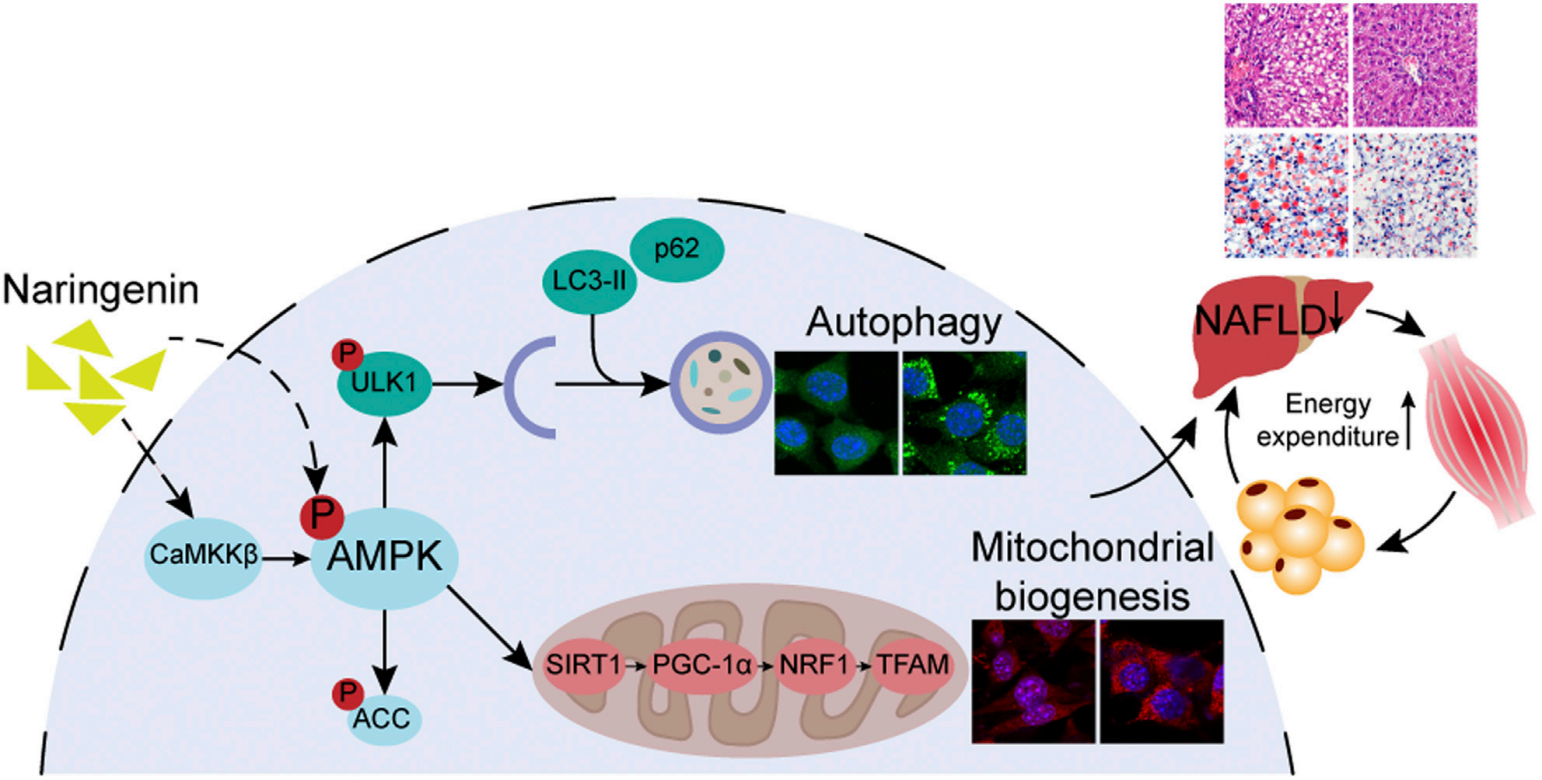
GRAPHICAL ABSTRACT

**FIGURE 1:**
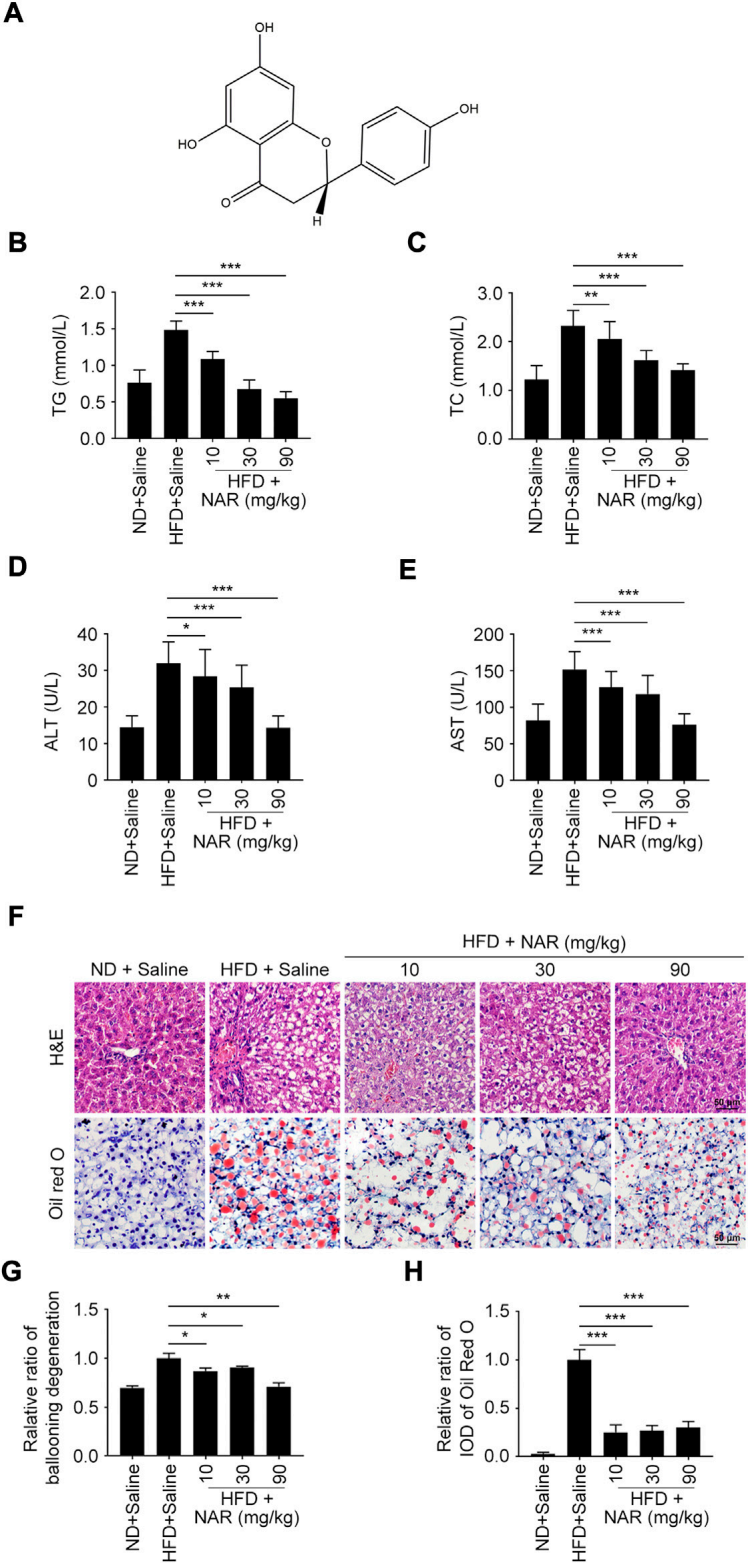
NAR attenuates HFD-induced NAFLD in rats. Male Sprague-Dawley rats aged 8 weeks were fed with an HFD or ND for 12 weeks. Then they were orally administered with NAR at 10, 30, or 90 mg/kg for 2 weeks *n* = 5 rats per group. **(A)** Chemical structure of NAR. **(B–E)** Serum level of TG, TC, ALT, and AST. **(F–H)** Representative H&E and oil red O staining of hepatic sections (the objective amplification 10×). Bars represent 50 μM. All values were expressed as the mean ± SD from three or more independent experiments. ***p < 0.001; **p < 0.01; *p < 0.05 vs. HFD group. Abbreviation: NAR, naringenin; NAFLD, nonalcoholic fatty liver disease; ND, normal diet; HFD, high-fat diet; TG, triglyceride; TC, total cholesterol; ALT, alanine aminotransferase; AST, aspartate aminotransferase; IOD, integrated optical density; H&E, hematoxylin and eosin.

NAR (NAR, 4′,5,7-trihydroxy flavanone, Figure 1A) is a flavanone and the aglycone of naringin. NAR is abundant in a lot of fruits, vegetables, and nuts, such as lemon, grapefruit, oranges, and tomatoes. NAR has been reported to modulate several biological processes related to NAFLD including energy balance, lipid and glucose metabolism, inflammation, and oxidative stress (Naeini et al., 2020). However, whether or not NAR attenuates NAFLD by activating adenosine 5′-monophosphate (AMP)-activated protein (AMPK), the key regulator in energy balance, is unclear. Furthermore, most *in vivo* studies of NAR were conducted in large doses (50–100 mg/kg) and long-term use (4–12 weeks) (Den Hartogh and Tsiani, 2019). Additionally, few studies of NAR simultaneously employ cells from the liver, skeletal muscle, and adipose.

We established the high-fat diet (HFD)-induced NAFLD model in rats, and orally administered NAR at 10, 30, and 90 mg/kg for 2 weeks. The effects and mechanisms of NAR on lipid accumulation and energy expenditure were investigated by *in vitro* experiments.

## Materials and Methods

### Animal Experiment

The experiment was conducted following the Guidance on the Care and Use of Laboratory Animals (U.S. National Institutes of Health Publication No.80-23, revised in 1996) and approved by the Medical Ethics Committee of Sichuan University (No. K2020435). Male Sprague-Dawley rats aged 8 weeks were commercially obtained (Dossy, Chengdu, China). All together 25 rats were randomly divided into five groups: normal chow diet (ND) group, HFD group, NAR 10 mg/kg group, NAR 30 mg/kg group, and NAR 90 mg/kg group, with five rats per group. Rats of the ND group were fed with an ND (Dossy, Chengdu, China) for 12 weeks. Rats of the HFD group and NAR groups were fed with an HFD (60% fat calories, HFK Bioscience, Beijing, China) for 12 weeks (Lau et al., 2017). NAR (C_15_H_12_O_5_) with purity above 99% was commercially obtained from Herbest Biotechnology (Baoji, China). Rats of the NAR groups were orally administered with NAR daily for 2 weeks. Rats of the ND group and HFD group were orally administered with saline for 2 weeks. Rats continued their diet until being sacrificed. Serum and liver samples were obtained.

### Serum Assays

Serum triglyceride (TG), total cholesterol (TC), glutamic-oxaloacetic transaminase (AST), and glutamic-pyruvic transaminase (ALT) were measured using TG colorimetric assay kit based on glycerol phosphate oxidase-peroxidase method, TC colorimetric assay kit based on cholesterol oxidase-peroxidase method, ALT colorimetric assay kit, AST colorimetric assay kit, respectively (Jiancheng, Nanjing, China) (Zhao et al., 2020). The absorbance of all samples was measured at 510 nm with the multiskan FC microplate reader (Thermo Fisher, Waltham, MA, United States).

### Hematoxylin-Eosin Staining

Liver samples of rats were fixed with 4% paraformaldehyde (PFA, Sangon, Shanghai, China) in phosphate buffer saline (PBS) at 4°C for 16 h. Then samples were dehydrated, embedded in paraffin, sectioned into 6  μm slides, and stained using 0.5% hematoxylin and eosin (Zhao et al., 2020). All images were pictured using an optical microscope (Zeiss, Jena, Germany) and analyzed by Image-Pro Plus software (Media Cybernetics, Rockville, MD, United States).

### Cell Culture

L02 cells, 3T3-L1 preadipocytes, and C2C12 myoblasts were obtained from the American Type Culture Collection. Huh-7 cells were a kind gift from Prof. Lang Bai of West China Hospital, Sichuan University. L02 cells were cultured with Roswell Park Memorial Institute (RPMI) 1640 medium (HyClone, Logan, UT, United States) supplemented with 10% fetal bovine serum (FBS) and 1% penicillin/streptomycin. Huh-7 cells were cultured with Dulbecco’s Modified Eagle medium (DMEM, HyClone, Logan, UT, United States) supplemented with 10% FBS and 1% penicillin/streptomycin.

To differentiate into insulin resistant 3T3-L1 adipocytes, 3T3-L1 preadipocytes were induced with the DMEM supplemented with 10% NBCS, 1 µM dexamethasone, 0.5 mM 3-isobutyl-1-methylxanthine, and 1 μg/ml insulin for 48 h. Then the medium was changed to the DMEM supplemented with 10% NBCS and 1 μg/ml insulin for 48 h. Then the medium was changed to the DMEM supplemented with 10% NBCS and 1% penicillin/streptomycin. More than 90% of 3T3-L1 preadipocytes differentiated to adipocytes after induction for 8–10 days, which showed a fat cellular phenotype filled with lipid droplets.

C2C12 myoblasts were cultured in the DMEM medium supplemented with 10% FBS and 1% penicillin/streptomycin. C2C12 myoblasts were treated with DMEM medium supplemented with 2% horse serum (HyClone, Logan, UT, United States) for 48 h to induce differentiation into insulin resistant C2C12 myotubes. Mononucleated C2C12 myoblasts fused to form multinucleated C2C12 myotubes after being induced for 4–6 days. All cells were maintained at 37°C in a humidified atmosphere containing 5% CO_2_.

### Oil Red O Staining

Fresh liver samples were embedded in optimal cutting temperature compound (O.C.T.) and dissected into 10 μM sections. Then sections were stained with oil red O solution (3 mg/ml) and rinsed as described (Yao et al., 2016). L02 and Huh-7 cells were treated with sodium oleate at 125 µM for 24 h to establish a NAFLD cell model. Then cells were fixed with 4% PFA for 20 min, stained with freshly diluted oil red O staining solution at room temperature (RT) for 45 min, and rinsed as described (Chu et al., 2019).

### Glucose Uptake Assay

3T3-L1 adipocytes and C2C12 myotubes were treated with metformin (Sangon Bio-Tech, Shanghai, China) as the positive control, phloretin (BioVision, Milpitas, CA, United States) as the negative control, and NAR for 4 h. Glucose uptake was measured using the 2-[N-(7-nitrobenz-2-oxa-1,3-diazol-4-yl) amino]-2-deoxy-glucose (2-NBDG) glucose uptake assay kit (BioVision, Milpitas, CA, United States) (Waters et al., 2019). Images were taken by fluorescence microscope (Zeiss, Jena, Germany). The fluorescence intensity of each photograph was measured by Image-Pro Plus software (Media Cybernetics, Rockville, MD, United States). The drug-free group was used as a control. The relative ratio of each group was obtained after normalization.

### ATP Content Assay

3T3-L1 adipocytes and C2C12 myotubes were treated with NAR for 24 h. Total ATP content was measured using an ATP colorimetric assay kit (BioVision, Milpitas, CA, United States) (Jian et al., 2017). The absorbance was measured at 570 nm with the multiskan FC microplate reader (Thermo Fisher, Waltham, MA, United States).

### Western Blotting Assay

As for rat livers, 100 mg freshly frozen samples were cut into small pieces, lysed using 400 μl tissue lysate including 4 μl phenylmethylsulfonyl fluoride, and ground with homogenizer on ice. Then samples were centrifuged at 10,000 rpm for 5 min at 4°C. The supernatant was collected. All together 50 μl 5 × SDS-PAGE sample loading buffer was added to the supernatant and heated at 98°C for 20 min. As for cellular samples, cells were washed twice with PBS, harvested, and centrifuged at 1,500 rpm for 2 min at 4°C. Then samples were resuspended in the 1 × SDS-PAGE sample loading buffer, and heated at 98°C for 10 min. Proteins were separated on a 10% SDS-polyacrylamide gel and transferred to a polyvinylidene fluoride membrane. Transferred membranes were blocked with 5% skim milk in Tris-buffered saline with 0.1% Tween 20 and then incubated with the primary antibody at RT for 2 h, including p-AMPK (Thr172, Cell signaling, Beverly, MA, United States), AMPKα (Cell signaling, Beverly, MA, United States), p-ACC (Ser79, Cell signaling, Beverly, MA, United States), AAC (Cell signaling, Beverly, MA, United States) CaMKKβ (Sango, Shanghai, China), LKB1 (Sango, Shanghai, China), p-LKB1 (Thr189, Sango, Shanghai, China), PGC1α (Proteintech, Rosemont, IL, United States), NRF1 (Proteintech, Rosemont, IL, United States), TFAM (Proteintech, Rosemont, United States), SIRT1 (Santa Cruz, Santa Cruz, CA, United States), p-ULK1 (Ser555, Cell signaling, Beverly, MA, United States), ULK1 (Cell signaling, Beverly, MA, United States), LC3 (MBL, Nagoya, Japan), p62 (MBL, Nagoya, Japan), and GAPDH (Santa Cruz, Santa Cruz, CA, United States). Then membranes were washed with Tris-buffered saline with 0.1% Tween 20 and incubated with horseradish peroxidase-conjugated secondary antibody (Sigma, St-Louis, MI, United States) at RT for 1 h (Zhao et al., 2020). Signals were visualized with an enhanced chemiluminescence kit (Mei5Bio, Beijing, China). Images were documented by a chemiluminescence imager (Clinx, Shanghai, China). The gray value of each band is analyzed by ImageJ (Bethesda, MD, United States). The drug-free group was used as a control. The relative ratio is obtained after normalization.

### Immunofluorescence Assay

Cells were seeded on coverslips in 24-well plates and treated with NAR as indicated. Then cells were fixed with 4% PFA for 20 min and permeabilized with 0.5% Triton X-100 for 15 min. Cells were blocked with 4% bovine serum albumin for 1 h, cultured with the primary antibody against LC3 (MBL, Nagoya, Japan) at RT for 2 h, and incubated with secondary antibody conjugated to fluorescein isothiocyanate (Invitrogen, Carlsbad, CA, United States) at RT for 1 h (Wang et al., 2013). The nucleus was stained with 0.5 μg/ml Hoechst 33,258 (Sigma, St-Louis, MI, United States) for 3 min. Images were acquired by confocal microscopy (Zeiss, Oberkochen, Germany). The fluorescence intensity of each image was measured by Image-Pro Plus software (Media Cybernetics, Rockville, MD, United States). The drug-free group was used as a control, and the relative ratio of each group was obtained after normalization.

### Mitotracker Orange Staining

Cells were seeded on coverslips in 24-well plates and treated with NAR as indicated. Cells were stained with 250 nM mitotracker orange probe staining solution (Invitrogen, Carlsbad, CA, United States) for 30 min (Shiao et al., 2000). Then cells were fixed with 4% PFA for 20 min and permeabilized with 0.5% Triton X-100 for 15 min. The nucleus was stained with Hoechst 33258 (Sigma, St-Louis, MI, United States) for 3 min. Images were pictured by confocal microscopy (Zeiss, Oberkochen, Germany). The fluorescence intensity of each photograph was measured by Image-Pro Plus software (Media Cybernetics, Rockville, MD, United States). The drug-free group was used as a control. The relative ratio of each group was obtained after normalization.

### Molecular Docking

The crystal structure of a CBS domain pair from the regulatory gamma1 (AMPKγ1) subunit of human AMPK in complex with AMP (PDB code: 2UV6, resolution: 2.00 Å) was downloaded from the RCSB protein data bank (http://www.rcsb.org/) and used as the receptor structure for molecular docking. 3D structure of NAR (PubChem Substance ID: 24900390, CAS: 67604-48-2) was obtained from PubChem (https://pubchem.ncbi.nlm.nih.gov/), converted to MOL2 format with PyMOL 2.4 software (Delano Scientific, San Carlos, CA, United States), and used as ligand structure for molecular docking. GetBox Plugin, a plugin of PyMOL 2.4 software, was used to obtain the active site for molecular docking. LeDock 1.0 software (version 1.0, http://lephar.com) was used for molecular docking (Liu et al., 2018). The binding pocket was set at 3.9, 25.0, 15.9, 31.6, 22.2, and 38.7, and the remaining parameters as default. PyMOL software was used to analyze the docking results.

### Statistical Analysis

All experiments were performed in triplicate and data were expressed as the mean ± SD. All data were analyzed with the Student’s *t*-test with GraphPad Prism 7.0 (GraphPad, La Jolla, CA, United States). *p* values less than 0.05 were considered statistically significant.

## Results

### Naringenin Attenuates HFD-Induced Non-Alcoholic Fatty Liver Disease in Rats

Serum levels of TG, TC, ALT, and AST of HFD-induced NAFLD rats were dose-dependently decreased by NAR at 10, 30, and 90 mg/kg (Figures 1B–E). As detected by H&E and oil red O staining, NAR alleviated ballooning degeneration and lipid accumulation in the livers of NAFLD rats (Figures 1F–H).

### Naringenin Attenuates Lipid Accumulation in L02 and Huh-7 Cells, Enhances the Glucose Uptake and Decreases the ATP Content in 3T3-L1 Adipocytes and C2C12 Myotubes

**FIGURE 2:**
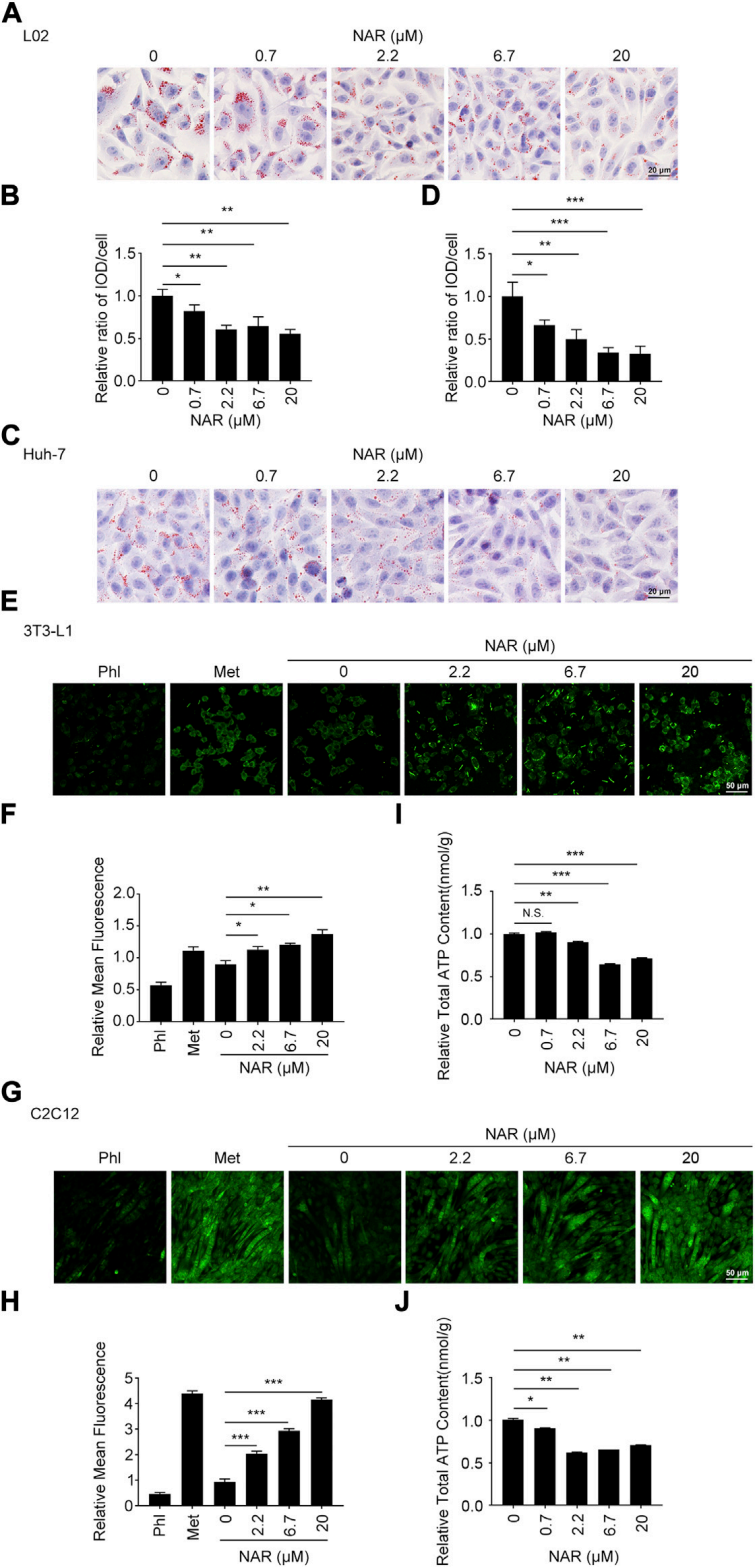
NAR alleviates lipid accumulation in L02 and Huh-7 cells, enhances glucose uptake, and decreases the ATP content in 3T3-L1 adipocytes and C2C12 myotubes. Sodium oleate-induced L02. **(A,B)** and Huh-7 cells **(C,D)** were treated with NAR (0, 0.7, 2.2, 6.7, and 20 μM) for 24 h. Lipid accumulation was visualized using oil red O staining (the objective amplification 20×). 3T3-L1 adipocytes **(E,F)** and C2C12 myotubes **(G,H)** were treated with NAR (0, 2.2, 6.7, and 20 μM), phloretin (100 μM), or metformin (2.5 mM) for 4 h. Glucose uptake was detected by 2-NBDG staining and photographed by fluorescence microscopy (the objective amplification 10×). The ATP content of 3T3-L1 adipocytes **(I)** and C2C12 myotubes **(J)** was determined using an ATP calorimetric assay kit. Bars represent 20 μM **(A,C)**, 50 μM **(E,G)**. All values were expressed as the mean ± SD from three or more independent experiments. ***p < 0.001; **p < 0.01; *p < 0.05 *vs*. untreated cells. Abbreviation: NAR, naringenin; Phl, phloretin; Met, metformin; 2-NBDG, 2-[N-(7-nitrobenz-2-oxa-1,3-diazol-4-yl) amino]-2-deoxy-glucose.

L02 and Huh-7 cells were induced by sodium oleate to establish the NAFLD cell model. As detected by oil red O staining, NAR significantly inhibited lipid accumulation in L02 (Figures 2A,B) and Huh-7 (Figures 2C,D) cells in a dose-dependent manner. As detected by 2-NBDG staining, NAR increased glucose uptake in both 3T3-L1 adipocytes (Figures 2E,F) and C2C12 myotubes (Figures 2G,H). NAR also decreased the total ATP content in both cells (Figures 2I,J).

### Naringenin Activates the CaMKKβ/AMPK/ACC Pathway and Enhances Mitochondrial Biogenesis of 3T3-L1 Adipocytes and C2C12 Myotubes

**FIGURE 3:**
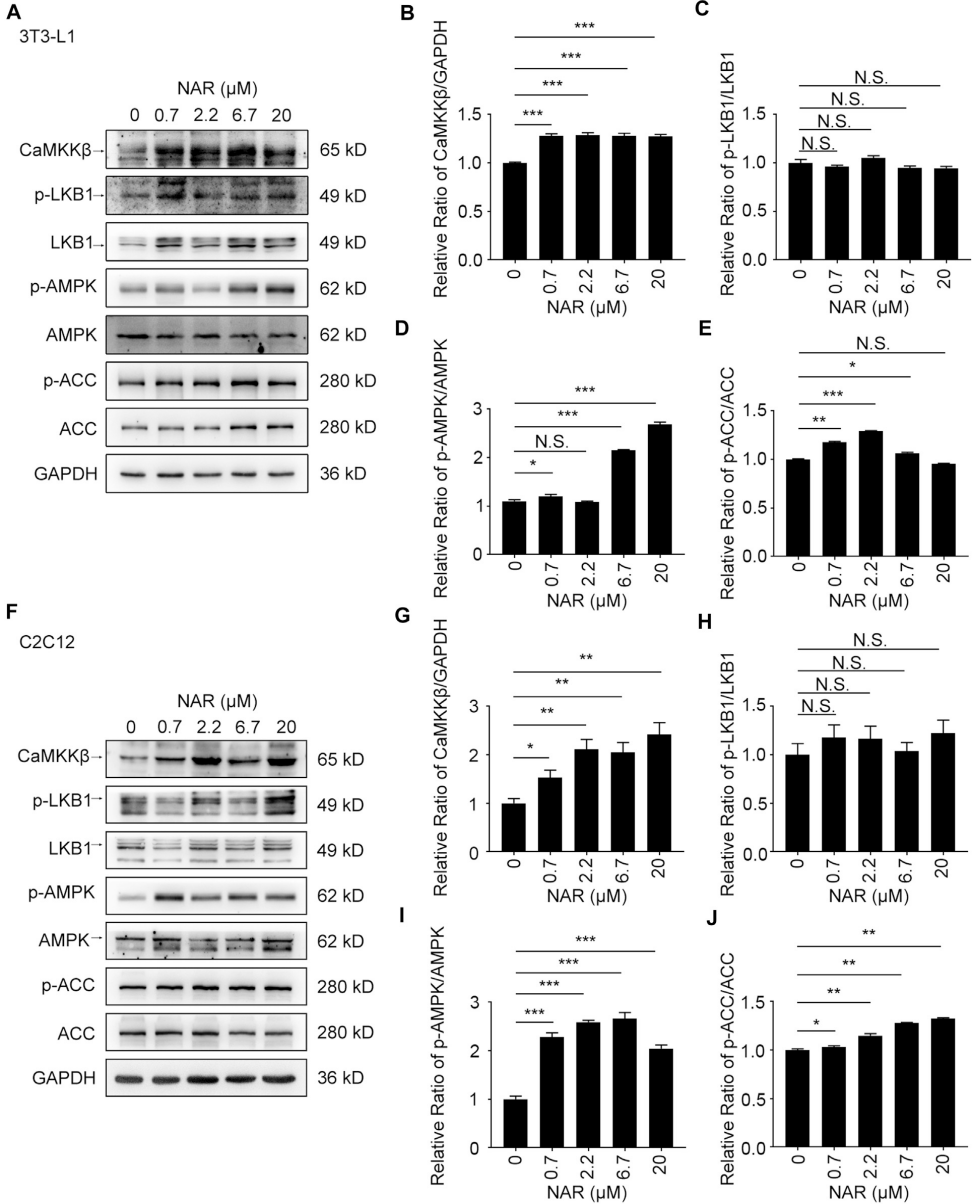
NAR activates the CaMKKβ/AMPK/ACC pathway in 3T3-L1 adipocytes and C2C12 myotubes. The expressions of CaMKKβ, p-LKB1, LKB1, p-AMPK, AMPK, p-ACC, and ACC of 3T3-L1 adipocytes **(A–E)** and C2C12 myotubes **(F–J)** after treatment of NAR were measured by Western blotting. All values were expressed as the mean ± SD from three or more independent experiments. ***p < 0.001; **p < 0.01; *p < 0.05 *vs*. untreated cells.; Abbreviation: NAR, naringenin; CaMKKβ, calmodulin-dependent protein kinase β; LKB1, liver kinase B1; p-LKB1, phosphor-LKB1; AMPK, AMP-activated protein kinase; p-AMPK, phosphor-AMPK; ACC, acetyl-CoA carboxylase 1; p-ACC, phosphor-ACC.

**FIGURE 4:**
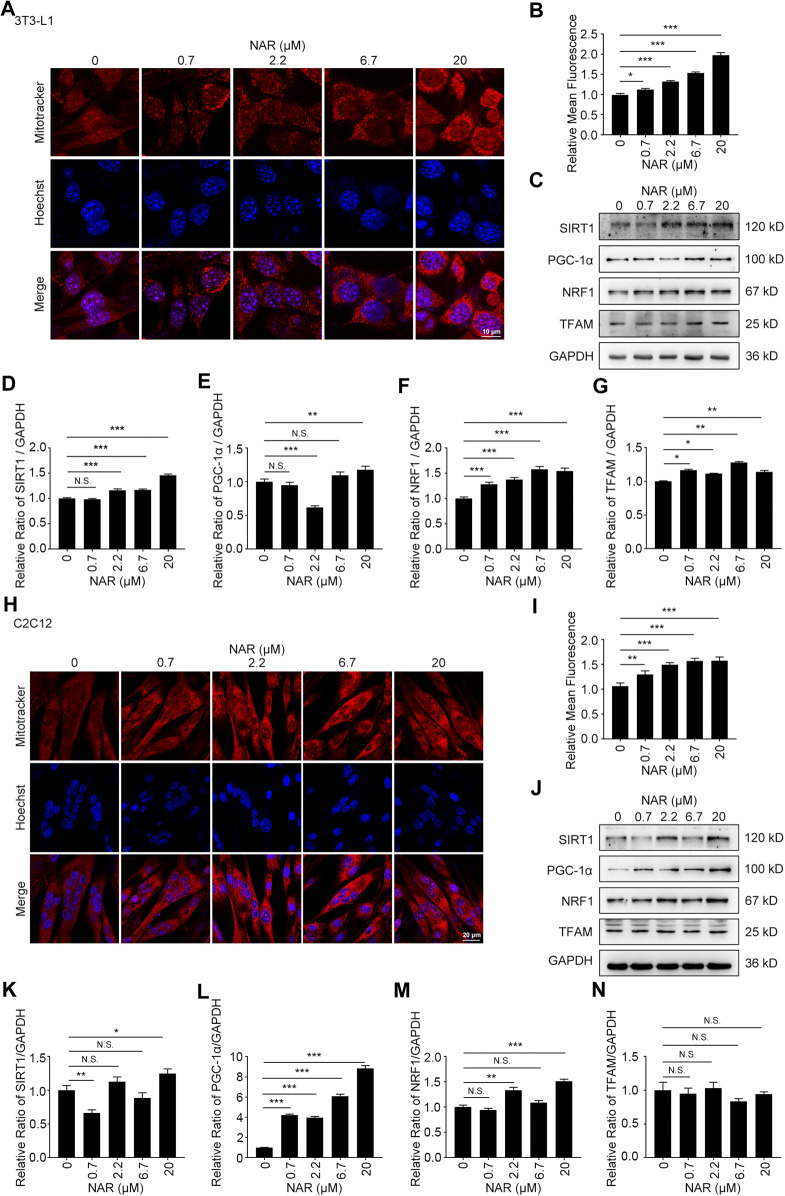
NAR promotes mitochondrial biogenesis of 3T3-L1 adipocytes and C2C12 myotubes. The mass of mitochondria in 3T3-L1 adipocytes (**(A,B)**, the objective amplification 63×) and C2C12 myotubes (**(H,I)**, the objective amplification 20×) after treatment of NAR was detected by mitotracker orange staining (red). The expression of SIRT1, PGC-1α, NRF1, and TFAM in 3T3-L1 adipocytes **(C–G)** and C2C12 myotubes **(J–N)** was determined by Western blotting. Bars represent 10 μM **(A)**, 20 μM **(H)**. All values were expressed as the mean ± SD from three or more independent experiments. ***p < 0.001; **p < 0.01; *p < 0.05 vs. untreated cells.; Abbreviation: NAR, naringenin; SIRT1, sirtuin1; PGC-1α, peroxisome proliferator-activated receptor coactivator 1α; NRF1, nuclear respiratory factor 1; TFAM, mitochondrial transcription factor A.

As detected by Western blotting, NAR increased the expression of CaMKKβ, promoted the phosphorylation of AMPK and ACC in 3T3-L1 adipocytes (Figures 3A–E) and C2C12 (Figures 3F–J). NAR significantly increased the expression of CaMKKβ in both cells (Figures 3A,B,F,G). Interestingly, the relative ratio of p-LKB1/LKB1 was not changed by NAR in both cells (Figures 3A,C,F,H ). As detected by mitotracker orange staining, NAR dose-dependently promoted the mitochondrial biogenesis in 3T3-L1 adipocytes (Figures 4A,B) and C2C12 myotubes (Figures 4H,I). NAR increased the expression of SIRT1 in 3T3-L1 adipocytes (Figures 4C,D) and C2C12 myotubes at 20 μM, and decreased the expression of SIRT1 at 0.7 μM (Figures 4J,K). Interestingly, NAR only increased the expression of PGC1α in 3T3-L1 adipocytes at 20 μM, but decreased the expression of PGC1α at 2.2 μM (Figures 4C,E). Moreover, NAR dose-dependently increased the expression of PGC1α in C2C12 myotubes at 0.7, 2.2, 6.7, and 20 μM (Figures 4J,L). NAR increased the expression of NRF1 in both cells (Figures 4C,F,J,M). NAR increased the expression of TFAM in 3T3-L1 adipocytes (Figures 4C,G), but not in C2C12 myotubes (Figures 4J,N).

### Naringenin Induces Autophagy and Promotes the Initiation of Autophagy in Autophagic Flux in 3T3-L1 Preadipocytes and C2C12 Myoblasts

**FIGURE 5:**
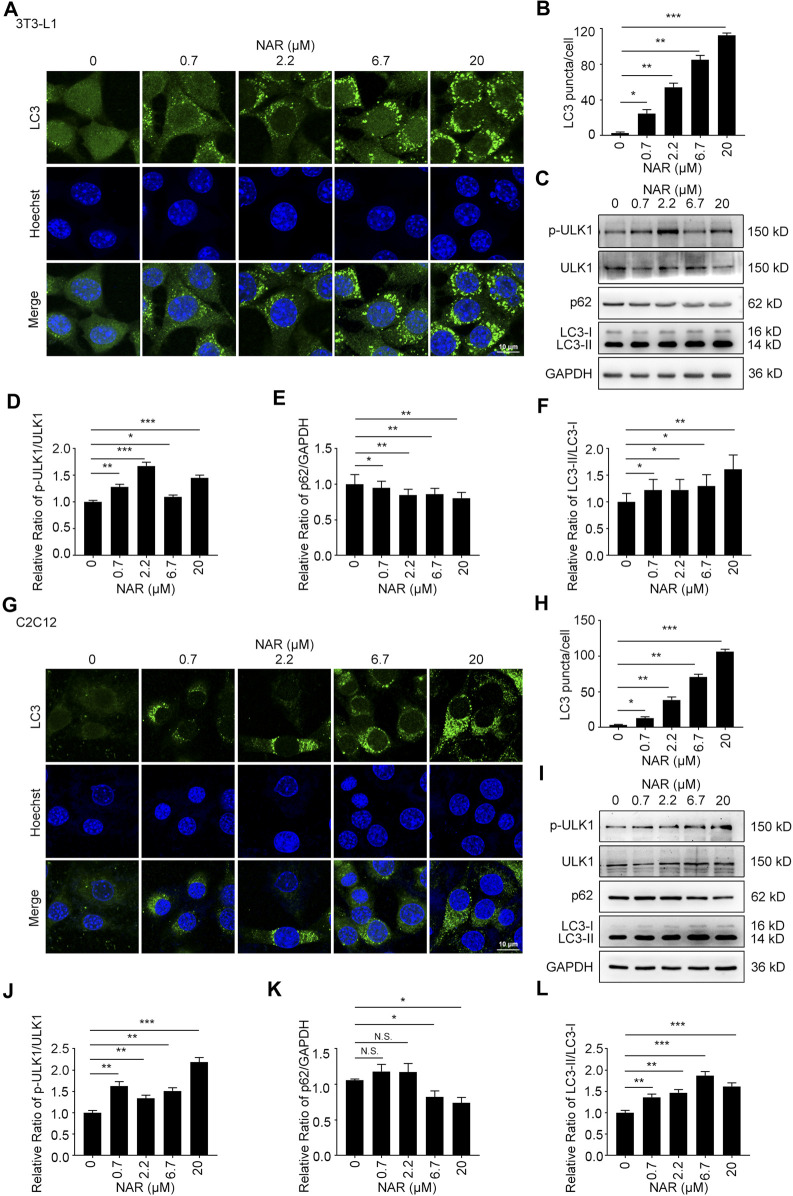
NAR induces autophagy in 3T3-L1 preadipocytes and C2C12 myoblasts. LC3 in 3T3-L1 preadipocytes **(A,B)** and C2C12 myoblasts **(G,H)** were detected by immunofluorescence (endogenous LC3 puncta, green), and pictured by confocal microscopy (the objective amplification 63×). The expression of p-ULK1, ULK1, p62, and LC3 in 3T3-L1 preadipocytes **(C–F)** and C2C12 myoblasts **(I–L)** was detected by Western blotting. Bars represent 10 μM. All values were expressed as the mean ± SD from three or more independent experiments. ***p < 0.001; **p < 0.01; *p < 0.05 *vs*. untreated cells.; Abbreviation: NAR, naringenin; ULK1, unc-51 like autophagy activating kinase 1; p-ULK1, phosphor-ULK1; p62, ubiquitin-binding protein p62; LC3, microtubule-associated protein 1A/1B-light chain 3.

**FIGURE 6:**
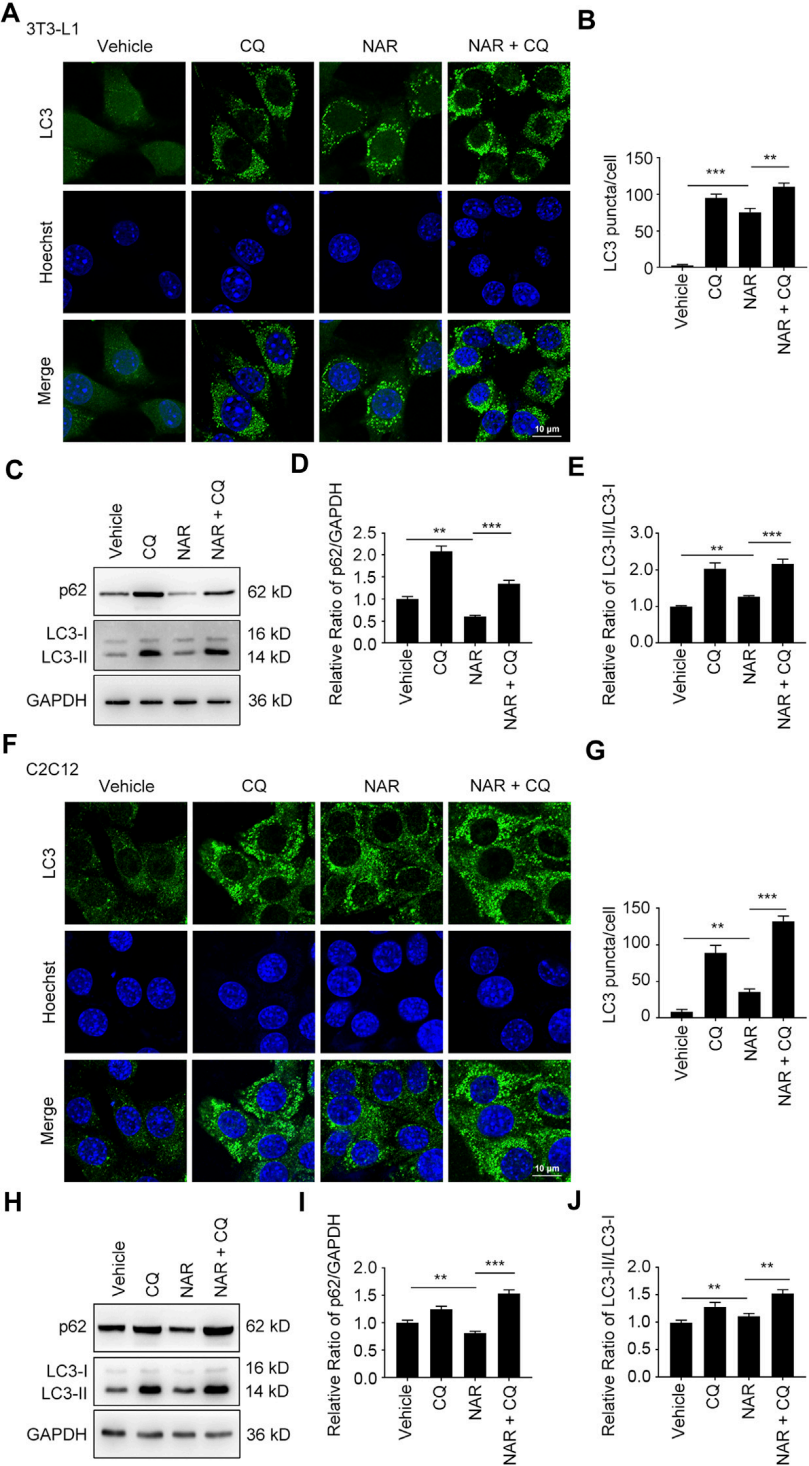
NAR promotes the initiation of autophagy in the autophagic flux of 3T3-L1 preadipocytes and C2C12 myoblasts. LC3 in 3T3-L1 preadipocytes **(A,B)** and C2C12 myoblasts **(F,G)** after treatment of NAR or in combination with CQ were detected by immunofluorescence (green), and pictured by confocal microscopy (the objective amplification 63×). The expression of p62 and LC3 in 3T3-L1 preadipocytes **(C–E)** and C2C12 myoblasts **(H–J)** was detected by Western blotting. Bars represent 10 μM. All values were expressed as the mean ± SD from three or more independent experiments. ***p < 0.001; **p < 0.01; *p < 0.05 vs. untreated cells or as indicated. Abbreviation: NAR, naringenin; CQ, chloroquine.

NAR dose-dependently increased endogenous LC3 puncta in both cells (Figures 5A,B,G,H). NAR promoted the phosphorylation of ULK1 in 3T3-L1 preadipocytes (Figures 5C,D) and C2C12 myoblasts (Figures 5I,J). NAR decreased the expression of p62 and increased the ratio of LC3-II/LC3-I in both cells (Figures 5C–F,I–L). Chloroquine (CQ), the inhibitor of autophagic flux by inhibiting autophagosome-lysosome fusion, was employed. The combination of CQ and NAR enhanced the LC3 puncta and the expression of p62 than NAR alone in 3T3-L1 preadipocytes (Figures 6A–D) and C2C12 myoblasts (Figures 6F–I). The combination of NAR and CQ also resulted in increased LC3-II turnover in both cells (Figures 6C,E,H,J).

### Naringenin Inhibits Autophagy in Non-Alcoholic Fatty Liver Disease rat Livers, 3T3-L1 Adipocytes, and C2C12 Myotubes

**FIGURE 7:**
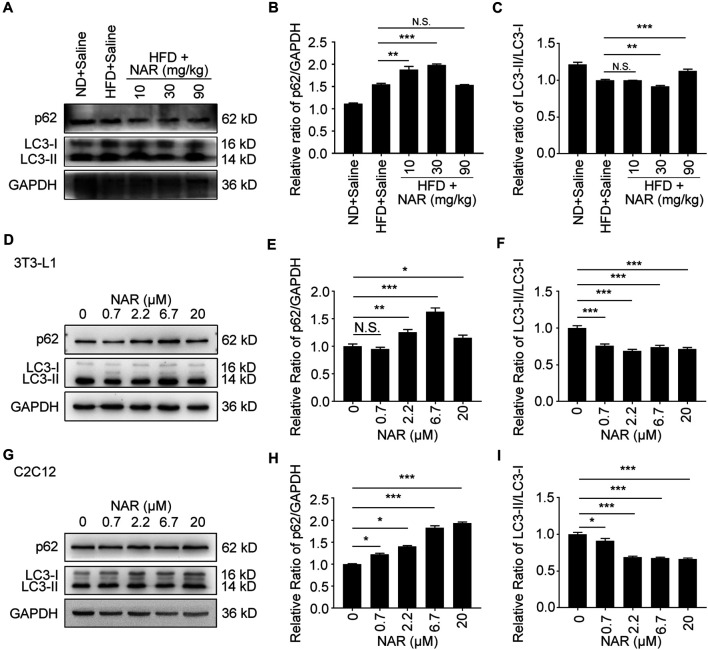
NAR decreases autophagy in livers of HFD-induced NAFLD rats, 3T3-L1 adipocytes, and C2C12 myotubes. The expression of p62 and LC3 in the HFD-induced NAFLD rat livers **(A–C)**, 3T3-L1 adipocytes **(D–F)**, and C2C12 myotubes **(G–I)** after treatment of NAR was detected by Western blotting. All values were expressed as the mean ± SD from three or more independent experiments. ***p < 0.001; **p < 0.01; *p < 0.05 *vs*. untreated cells.; Abbreviation: NAR, naringenin; NAFLD, nonalcoholic fatty liver disease; HFD, high-fat diet.

Interestingly, NAR decreased the expression of p62 in NAFLD rat livers at 30 and 90 mg/kg, decreased LC3-II/LC3-I at 30 mg/kg but increased it at 90 mg/kg (Figures 7A–C **).** NAR increased the expression of p62 in 3T3-L1 adipocytes (Figures 7D,E ) and C2C12 myotubes (Figures 7G,H). NAR dose-dependently decreased the ratio of LC3-II/LC3-I in both cells (Figures 7D,F,G,I).

### Naringenin Plays Effects by Binding to AMPKγ1

**FIGURE 8:**
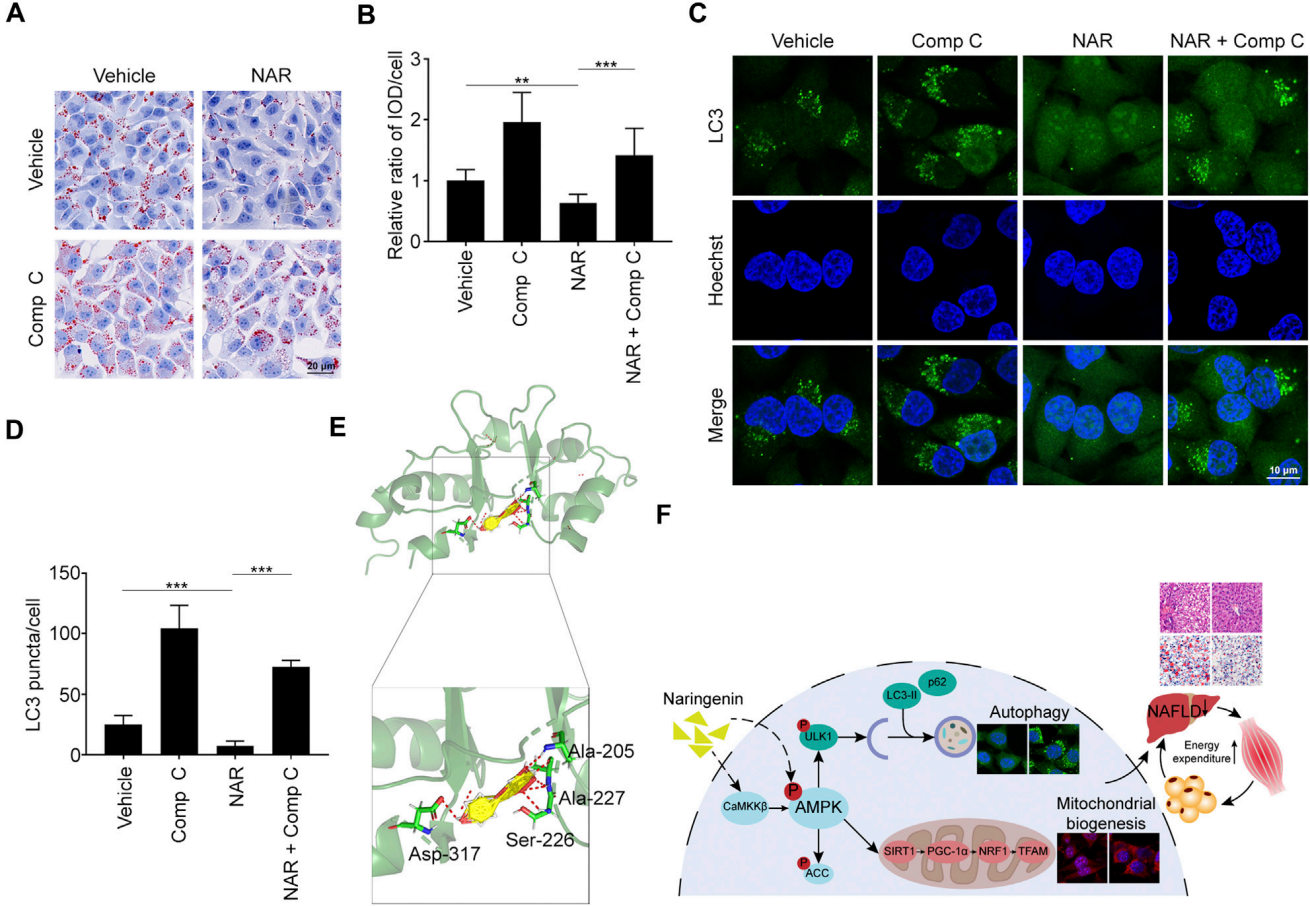
NAR exerts effects by binding to AMPKγ1. Sodium oleate-induced L02 cells were treated with NAR (6.7 μM) alone or in combination with Comp C (4 μM) for 24 h. **(A,B)** Lipid accumulation was detected using oil red O staining (the objective amplification 20×). **(C,D)** Endogenous LC3 in L02 cells were detected with immunofluorescence (green) (the objective amplification 63×). **(E)** Docking of NAR at the active sites of AMPKγ1 was performed by LeDock software. The red dotted line indicated a hydrogen bond. **(F)** The schematic diagram of our study. Bars represent 20 μM **(A)**, 10 μM **(C)**. All values were expressed as the mean ± SD from three or more independent experiments. ***p < 0.001; **p < 0.01; *p < 0.05 vs. untreated cells or as indicated. Abbreviation: NAR, naringenin; Comp C, Compound C; IOD, integrated optical density; Ala, Alanine; Ser, Serine; Asp, Aspartic acid; ACC, acetyl-CoA carboxylase 1; AMPK, AMP-activated protein kinase; CaMKKβ, calmodulin-dependent protein kinase β; LC3, microtubule-associated protein 1A/1B-light chain 3; NRF1, nuclear respiratory factor 1; PGC-1α, peroxisome proliferator-activated receptor coactivator 1α; p62, ubiquitin-binding protein p62; SIRT1, NAD-dependent protein deacetylase sirtuin-1; TFAM, mitochondrial transcription factor A; ULK1, Unc-51 like autophagy activating kinase 1.

Compound C (Comp C), an AMPK inhibitor, was employed. NAR inhibited lipid accumulation in sodium oleate-treated L02 cells, which was reversed by Comp C (Figures 8A,B). NAR enhanced autophagy in sodium oleate-treated L02 cells, which was also reversed by Comp C (Figures 8C,D). The binding of NAR and AMPKγ was explored by LeDock through molecular docking. NAR formed hydrogen bonds with AMPKγ1 residues Ala205, Ala227, Ser227, and Asp317 (Figure 8E).

## Discussion

Plant extracts such as flavonoids (dihydromyricetin, etc.) or phenolic compounds have multiple pharmacological activities such as regulating glucose metabolism (Chen et al., 2020a), anti-inflammatory (Chen L. et al., 2018), and anti-metabolic diseases effects (Chen et al., 2020b; Yao et al., 2021). The drug delivery system is being improved to enhance their bioavailability (Chen et al., 2019). Our study demonstrated that NAR attenuates NAFLD at low doses for the short term. Current clinical trials of NAR mainly focus on NAR bioavailability, cardio-protection, and glucose metabolism regulation effects (Salehi et al., 2019). Flavonoids such as quercetin, hesperetin, and NAR exert similar pharmacological effects since they share similar A ring structures. Quercetin and hesperetin differ from NAR by hydroxyl group at the 3-position and a C2–C3 double bond (Narayana et al., 2001). After ten healthy volunteers consumed equal amounts of the three flavonoids, the plasma area under the curve (AUC)_0–48h_ for quercetin and hesperetin were similar, whereas the AUC_0–48h_ of NAR was higher. Thus, the relative bioavailability of NAR was higher than quercetin and hesperetin. Most studies of NAR’s effects for NAFLD on animal models were conducted in large doses (50–100 mg/kg) and (or) in a long time (4–12 weeks) (Naeini et al., 2020). Wang et al. reported that NAR attenuated hepatic lipid accumulation and inflammation in methionine and choline deficient diet-induced NAFLD mice at 100 mg/kg for 7 days (Wang Q. et al., 2020). We orally administered NAR at 10, 30, and 90 mg/kg to HFD-induced NAFLD rats for 2 weeks. NAR attenuated ballooning degeneration and lipid accumulation in the liver and decreased serum levels of TG, TC, AST, and ALT of NAFLD rats. We also observed that NAR attenuated lipid accumulation in L02 and Huh-7 cells at low concentration (0–20 μM) for 24 h. Therefore, our study confirmed the clinical significance of NAR for NAFLD at low doses and short-term, which suggested its potential use as a dietary supplement. Besides, our study confirmed the effects of NAR on cells from the liver (Huh-7 and L02 cells), skeletal muscle (C2C12 myoblasts and myotubes), and adipose (3T3-L1 preadipocytes and adipocytes). Our results showed that NAR increased glucose uptake in both 3T3-L1 adipocytes and C2C12 myotubes. NAR had been reported to inhibit α-glucosidase activity which delayed carbohydrate absorption (Priscilla et al., 2014). NAR was also reported to decrease the glucose uptake by inhibiting sodium-glucose co-transporters in the intestine (Li et al., 2006). These results indicated that NAR attenuates NAFLD through playing effects in major insulin target organs.

It’s our second major finding that NAR attenuates NAFLD through enhancing energy expenditure. Chronic energy imbalance is the common ground of metabolic disorders such as obesity, NAFLD, atherosclerosis, and T2DM (Roden and Shulman, 2019). It was reported that 3% NAR in the diet for 11 weeks significantly decreased the calorie intake and body weight of ovariectomy-associated NAFLD mice (Ke et al., 2015). We observed that NAR did not change the calorie intake and body weight of rats at low doses for the short term although NAR did attenuate the development of HFD-induced NAFLD (Supplementary Figure S1). The difference may be that we employed male rats while they employed female mice. The second reason may be the low-dose and short-term use in our study. According to our results, we hypothesized that NAR attenuates NAFLD through increasing energy expenditure. We further tested our hypothesis by *in vitro* experiments. NAR did increase the glucose uptake and decrease the ATP content of 3T3-L1 adipocytes and C2C12 myotubes. NAR in the aglycone of naringin, that, in turn, is reported to increase AMPK phosphorylation (Sui et al., 2018). Our results also demonstrated that NAR increased the phosphorylation of AMPK. AMPK-activated SIRT1/PGC-1α/NRF1/TFAM pathway plays key roles in maintaining the number of normal mitochondria in cells and promoting energy expenditure (Yu et al., 2019). Our results showed that NAR promoted the phosphorylation of AMPK and activated the SIRT1/PGC-1α/NRF1/TFAM pathway to enhance mitochondrial biogenesis of 3T3-L1 adipocytes and C2C12 myotubes. NAR was reported to exert similar effects in kidney cells, muscle cells, and cardiomyocytes (Kanno et al., 2006; Zygmunt et al., 2010; Yu et al., 2019). Since NAR enhances energy expenditure, it also may possess beneficial effects for metabolic disorders such as obesity, atherosclerosis, and T2DM.

Flavanones such as hesperidin and naringin have been demonstrated to reduce the expression of glucose transporter 2 (GLUT2) in the liver of diabetic mice (Jung et al., 2006; Hajiaghaalipour et al., 2015). NAR is the metabolite of naringin in humans (Chen T. et al., 2018). We assumed that naringenin had similar effects on the expression of GLUT2 with naringin. NAR has been reported to enhance the expression of GLUT4 in the livers of streptozotocin-induced diabetic rats (Singh et al., 2018).

As a famous energy sensor and regulator, AMPK plays a key role in energy expenditure (Shrikanth and Nandini, 2020). Inhibiting AMPK blocked the effects of NAR in improving insulin sensitivity and attenuating diabetes mellitus (Li et al., 2019). Most reports focus on NAR’s effects on the activity of AMPK, its regulation on the AMPK signaling pathway is poorly understood (Yu et al., 2019). NAR dose-dependently promoted the phosphorylation of AMPK and ACC in 3T3-L1 adipocytes and C2C12 myotubes. Interestingly, NAR dose-dependently increased the expression of CaMKKβ in both cells, while NAR did not change the ratio of p-LKB1/LKB1. Our findings suggested that NAR activated the CaMKKβ/AMPK/ACC pathway via a noncanonical LKB1-independent pathway. Comp C, the inhibitor of AMPKγ, abolished the effects of NAR on inhibiting lipid accumulation and enhancing autophagy in L02 cells. As explored by molecular docking, NAR formed hydrogen bonds with AMPKγ1 residues Ala205, Ala227, Ser227, and Asp317 at the site of AMPKγ1 binds to AMP (Day et al., 2007). Altogether, our results indicated that NAR may activate AMPK directly as AMP mimic or indirectly by activating CaMKKβ.

NAR was reported to reduce the activity of SIRT1 in cell-free histone deacetylase activity assay. NAR increased the expression and activity of SIRT1 in lipopolysaccharide-induced-THP-1 cells. These reports suggested that NAR may have a dual role in activating SIRT1. Our results showed that NAR did not increase the expression of SIRT1 at 0.7–6.7 μM, while the reason remains unclear. NAR binds to estrogen receptors (ER) α and β (Kuiper et al., 1998; Bulzomi et al., 2012), and acts as an estradiol mimic (Kim and Park, 2013). We speculate that NAR has multiple targets. NAR is mainly bound to AMPKγ1 to increase AMPK activity at low concentrations. NAR bound to ER to promote the expression of SIRT1 and increase mitochondrial biogenesis at higher concentrations. In the liver and muscle tissue of HFD-fed C57BL/6 J mice or primary human adipose tissue, NAR significantly increased the expression of PGC mRNA (Ke et al., 2015; Rebello et al., 2019). Our study showed that NAR significantly increased the expression of PGC-1α in C2C12 myotubes as detected by Western blotting but not in 3T3-L1 adipocytes. We speculated that NAR has different effects on skeletal muscle and adipose tissue.

Roles of autophagy in NAFLD remain controversial. Drugs against NAFLD by either increasing autophagy or inhibiting autophagy were reported (Chu et al., 2019; Ebrahimi et al., 2019; Ren et al., 2019). We observed that NAR induced autophagy and promoted the initiation of autophagy in autophagic flux in 3T3-L1 preadipocytes and C2C12 myoblasts. However, NAR inhibited autophagy in livers of HFD-induced NAFLD rats, 3T3-L1 adipocytes, and C2C12 myotubes. Therefore, we speculated that NAR regulated autophagy according to cellular status.

Our study has a couple of limitations. The binding of NAR and AMPKγ1 was explored by molecular docking and Comp C, which cannot demonstrate the direct binding of NAR and AMPKγ1. NAR regulated autophagy according to the cellular status whose mechanisms need further clarification.

In conclusion, we demonstrated that NAR attenuates NAFLD in short-term use of low doses by enhancing energy expenditure, which may be a candidate for NAFLD, even metabolic disorders such as obesity. The mechanisms involve regulating autophagy by directly or indirectly activating AMPK.

## Data Availability

The raw data supporting the conclusion of this article will be made available by the authors, without undue reservation.

## Glossary

ACC: acetyl-CoA carboxylase 1
Ala: alanine
ALT: alanine aminotransferase
AMPK: AMP-activated protein kinase
Asp: aspartic acid
AST: aspartate aminotransferase
AUC: area under the curve
CaMKKβ: calmodulin-dependent protein kinase β
Comp C: compound C
CQ: chloroquine
DMEM: Dulbecco’s modified Eagle medium
FBS: fetal bovine serum
GLUT2: glucose transporter 2
GLUT4: glucose transporter 4
H&E: hematoxylin eosin
HFD: high fat diet
IOD: integrated optical density
IR: insulin resistance
LKB1: liver kinase B1
LC3: microtubule-associated protein 1A/1B-light chain 3
Met: metformin
NAFLD: non-alcoholic fatty liver disease
NAR: naringenin
NBCS: newborn calf serum
ND: normal diet
NRF1: nuclear respiratory factor 1
O.C.T. compound: optimal cutting temperature compound
p-ACC: phosphor-ACC
p-AMPK: phosphor-AMPK
PGC-1α: peroxisome proliferator-activated receptor coactivator 1α
Phl: phloretin
p-LKB1: phosphor-LKB1
PMSF: phenylmethylsulfonyl fluoride
p-ULK1: phosphor-ULK1
PVDF: polyvinylidene fluoride
p62: ubiquitin-binding protein p62
RPMI: Roswell Park Memorial Institute
RT: room temperature
Ser: serine
SIRT1: NAD-dependent protein deacetylase sirtuin-1
TC: total cholesterol
TFAM: mitochondrial transcription factor A
TG: triglyceride
T2DM: type II diabetes mellitus
ULK1: Unc-51 like autophagy activating kinase 1
2-NBDG: 2-[N-(7-nitrobenz-2-oxa-1,3-diazol-4-yl) amino]-2-deoxy-glucose
