# Supplementary figures and images for: Naringenin Attenuates Non-Alcoholic Fatty Liver Disease by Enhancing Energy Expenditure and Regulating Autophagy via AMPK
Source: Front Pharmacol. 2021 Jun 7;12:687095. doi: 10.3389/fphar.2021.687095 (PMC8215389; doi:10.3389/fphar.2021.687095)

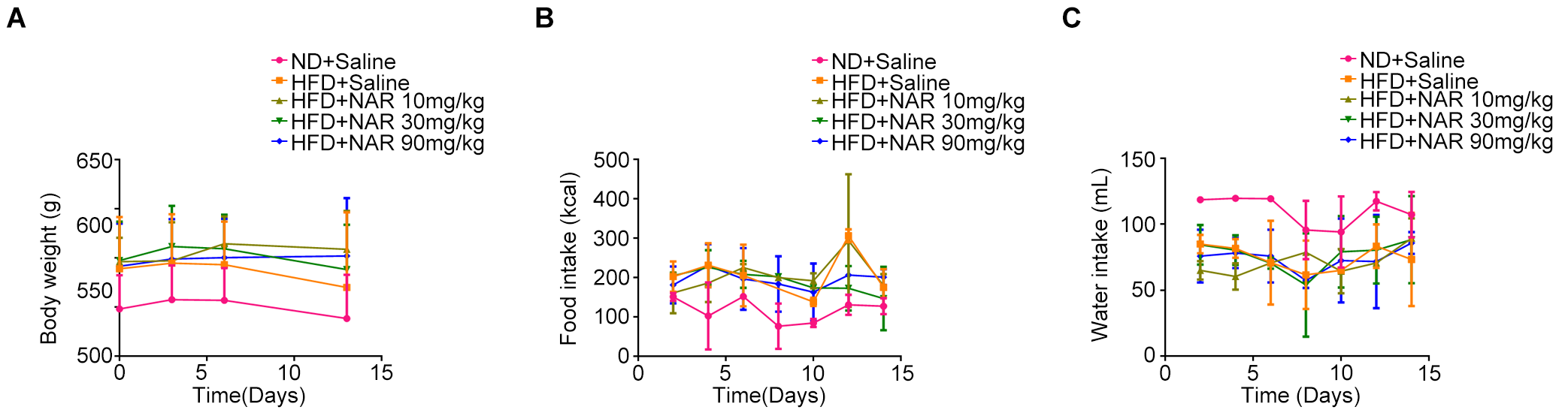

Supplement: Supplementary file 1 [file Image1.tif]
